# Extensive suprasternal dehiscence reconstruction with NPWT and advancement flaps following cardiac surgery

**DOI:** 10.1093/jscr/rjad623

**Published:** 2023-11-12

**Authors:** Boglarka Juhasz, Robert Tamas

**Affiliations:** Adult Cardiac Surgery Department, Gottsegen National Cardiovascular Center, Haller Street 29, 1096 Budapest, Hungary; Plastic Surgery Department, Hungarian Defense Forces Medical Centre, Robert K. sgt. 44, 1134 Budapest, Hungary

**Keywords:** DSWI, suprasternal dehiscence, wound complications following cardiac surgery, plastic surgery reconstruction

## Abstract

Treatment of suprasternal wound infection (SSWI) following cardiac surgery is not a clearly developed procedure. We report our female patient’s secondary SSWI treatment following bypass surgery. An obese female patient with unstable angina underwent an urgent, uneventful off-pump coronary artery bypass operation. An SSWI appeared within a week. After negative pressure wound therapy (NPWT), the sternum was rewired. In the previously irradiated territory of the left breast necrosis formed, a plastic surgeon reconstructed a defect. This procedure failed NPWT was restarted again, and a secondary reconstructive plastic surgery intervention was necessary. Despite extensive tissue mobilization, the central part of the reconstructive area necrotized, and we had to cover it with a split thickness skin mash graft. The irradiation therapy increases the incidence of suprasternal and/or sternal infection. It was possible to manage large soft tissue defects with bilateral and rotational advancement flaps.

## Introduction

Mediastinal wound infection following cardiac surgery is a rare but life-threatening complication. The incidence of deep sternal wound infection (DSWI) ranges between 0.4 and 5.1%. Despite appropriate antibiotic therapy (AB), the mortality rate is high at between 10 and 40% [1–5]. If DSWI occurs early and aggressive debridement is required, this means reopening of the wound, removal of the sternal wires and necrotic tissue, and sampling for bacteriology. Any delay in the diagnosis decreases the likelihood of sternum preservation. Earlier closed irrigation therapy was the treatment of choice [6], but, despite aggressive surgical interventions, the mortality rate was not decreased significantly. Since the 1990s, negative pressure wound therapy (NPWT) has been used worldwide as a first-line treatment for sternal wound infections [7, 8]. Superficial sternal wound infections (SSWIs) are quite frequent and less severe, with an incidence varying between 0.03 and 7.7% [9–11]. Superficial wound infections without extensive tissue loss due to several wound dressing changes and/or a minor NWPT system (e.g. PICO—single-use NPWT system, Smith & Nephew Healthcare Ltd, UK) usage have the potential to heal or, if necessary, be covered with half-thickness skin autografts. In the event of more extensive dehiscence after wide resection of the necrotic tissues and NPWT therapy, it was possible to close the defect with bilateral musculocutaneous advancement flaps [9]. We also use this technique for DSWI and SSWIs following wound preparation by NPWT. Our technique is reproducible, provides adequate arm and chest range of motion, and produces acceptable cosmetic results.

## Case presentation

**Figure 1:**
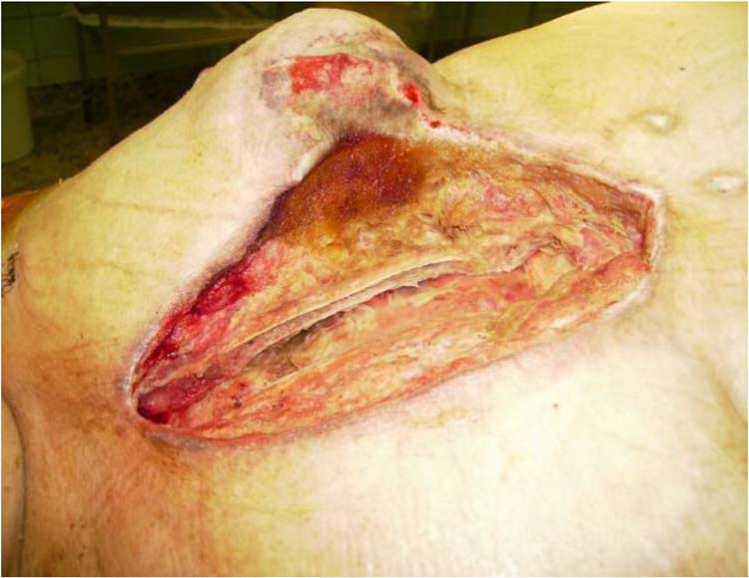
Operative view after debridement and NPWT.

**Figure 2:**
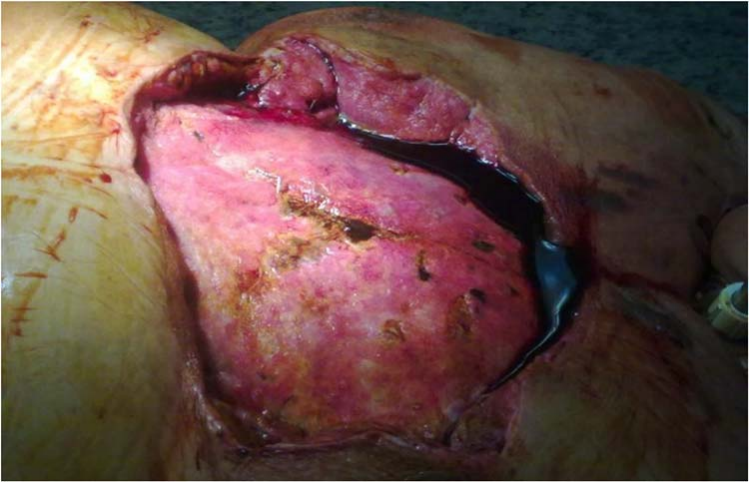
Extensive suprasternal dehiscence after sternal closure and NPWT.

A 67-year-old female patient with three vessel diseases and several co-morbidities (smoker, hypertensive, non-insulin-dependent diabetic) and unstable angina pectoris with good ejection fraction underwent an urgent, uneventful OPCAB operation in August 2010. A non-skeletonized left internal mammary artery (LIMA) was anastomosed to the left anterior descending coronary artery (LAD), and a saphenous vein graft was used for the anterior marginal coronary artery. The occluded right coronary artery was not suitable for graft implantation, so percutaneous coronary intervention (PCI) was performed on post-operative Day 4. Previously, the patient had left-sided breast cancer, which was treated with partial resection and irradiation therapy. Her BMI was 32, and the Parsonnet score was 14. SSWI appeared within a week and spread rapidly to the deep layers with the patient soon becoming septic. *Staphylococcus aures* was detected in the samples. After re-operation and aggressive debridement (sternal wire removal, necrotic tissue resection), wound infection was controlled by NPWT (continuous suction of 125Hgmm, KCI Medizinprodukte GmbH) (Fig. 1). Following multiple negative bacteriology samples and 3 weeks of NPWT, the sternum was rewired. Reconstruction of the suprasternal tissues was delayed because thick necrosis with a 5 × 6 cm diameter and an increasing tendency developed in the previously irradiated area of the left breast. Samples were taken from this area for histological examination and the wound was treated with NWPT for 2 weeks as an SSWI. As no malignancy formation was found in the histological samples, we reconstructed the suprasternal tissues with bilateral advancement flaps as in previous groups described elsewhere [9–15]. Three days after the procedure, severe bleeding occurred at the surgical site, the wound ruptured, and hypovolemic shock developed. After urgent re-operation and bleeding control, suprasternal NPWT therapy was resumed. Together with prolonged intravenous antibiotic (AB) treatment, this led to septic shock caused by pseudomembranous colitis. Enterococcus and *Proteus mirabilis* were cultured from repeated wound samples. After modification of intravenous AB therapy and 1 month in intensive care, an improvement was seen in the patient. Suprasternal dehiscence was too extensive (~20 × 25 cm) (Fig. 2) to be simply closed, so another reconstructive plastic surgery intervention was planned.

## Surgical techniques

**Figure 3:**
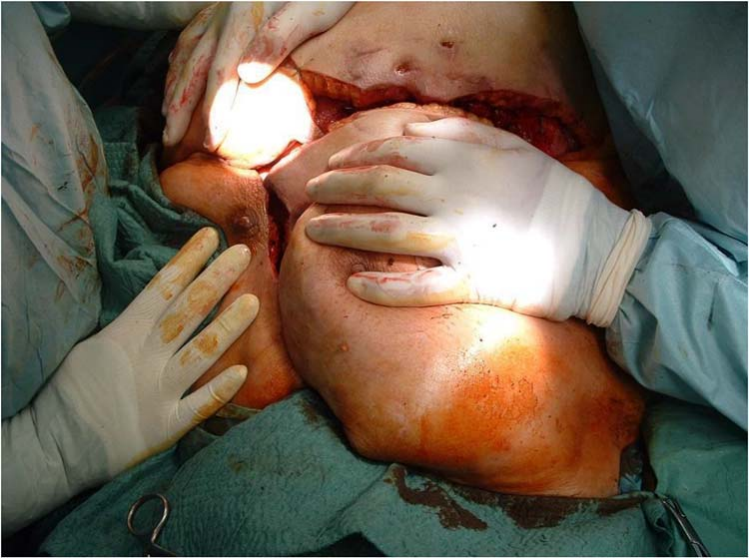
By sliding the advancement flaps towards the midline and cranially, approximately the upper two-thirds of the dehiscence were closed without any tension.

**Figure 4:**
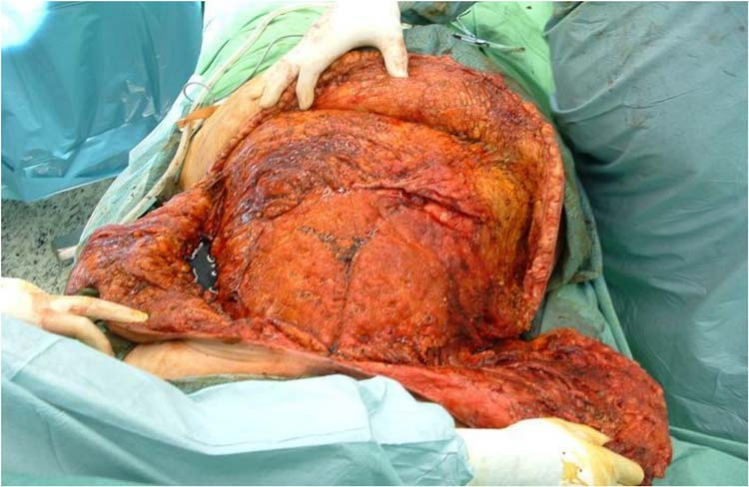
With the four advancement flaps, the huge dehiscence was fully covered with minimal tension.

**Figure 5:**
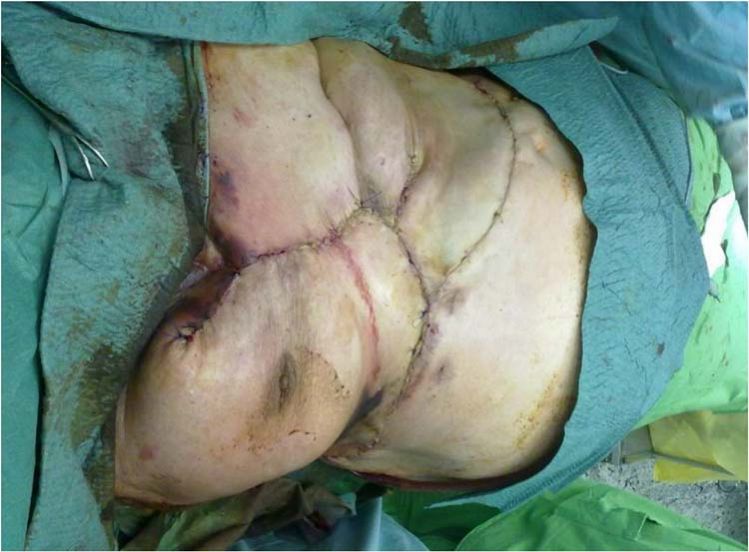
With this expanded tissue mobilization, we were able to cover the defect.

After debridement of the wound, we started under-preparing the wound edges around the dehiscence. Even with extensive preparation, we were only able to cover ~40% of the defect, so both lateral areas adjacent to the defect had to be mobilized bilaterally and medially. Because of the extensive suprasternal dehiscence, we decided to use not only bilateral musculocutaneous but also rotational and abdominal advancement flaps to cover the wound [12–15]. A lateral incision was made 4 cm below the right submammary fold, starting from the defect, and extending laterally along the lateral part of the chest wall to the posterior axillary line and the edge of the latissimus muscle. The right breast was undercut with the underlying pectoralis muscle to the axillary line. The previously under-prepared scarred base of the medial half of the right breast was incised, making this part of the flap more mobile. Under the left breast, the remaining stock was under-prepared with the underlying pectoral muscle, also laterally to the edge of the latissimus muscle and towards the axilla line, using an assisted incision. By sliding these two flaps towards the midline and cranially, approximately the upper two-thirds of the dehiscence were closed without any tension (Fig. 3). In the remaining lower third of the defect, the skin-fat layer of the abdominal wall was caudally under-prepared to form an ‘advancement flap’, which allowed a sliding of 5–6 cm towards cranial direction to cover the small dehiscence in the lower part of the sternum, a laterally mobilized fasciocutan flap was made under the left inframammary fold with a laterally made assistant and ‘cut back’ incision. With these four flaps, the huge dehiscence was fully covered with minimal tension (Fig. 4). The flaps were stitched with 2-0 Vicryl simple knot stitches to their bases, and the wound was closed with three layers of non-absorbable running sutures. With this expanded tissue mobilization, we were able to cover the defect (Fig. 5).

## Post-operative period

**Figure 6:**
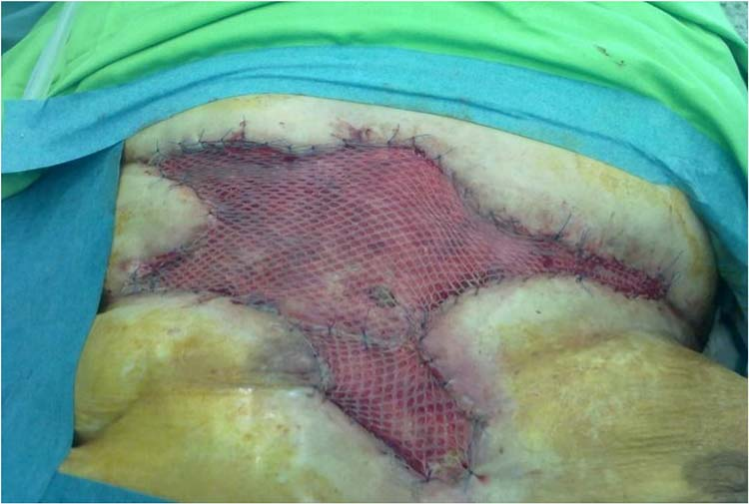
Two months after the second plastic surgery intervention, we had to cover the central dehiscence by applying a half-thickness skin autograft.

Despite the extensive tissue mobilization and untensioned bandages, the central part of the reconstruction was necrotized within a month. Two months after the second plastic surgery intervention, we had to cover this dehiscence by applying a half-thickness skin autograft (Fig. 6). The patient eventually left our department after 12 wound revisions, three plastic surgery reconstructions, 66 NPWT treatment days, 141 days in intensive care, and 4 months in our hospital. After all this, she was mentally and physically active. Two years ago, she died, the autopsy found nothing unusual.

## Discussion

Infections following cardiac surgery interventions will always be a pressing problem [1–5]. The management of mediastinal wound infections depends on sternal involvement but remains a controversial topic among cardiac surgeons. Earlier irrigation therapy [6] or different types of flap usage were the treatments of choice [12–16]. Since the 1990s, NPWT has been used worldwide as a first-line therapy in sternal wound infections [7, 8]. There are a lot of risk factors [1–5, 17]. Smoking habits, treatment for COPD, obesity, diabetes, corticosteroid therapy, pre- or post-operative IABP usage are the main preoperative risk factors. Bilateral ITA graft usage and pulmonary or ventilation problems are the most important intraoperative and post-operative risk factors [10, 16, 17]. Shafir *et al.* [18] identified that paramedian sternotomy is among the main risk factors in post-operative wound complication. Obesity has reached epidemic proportions worldwide and is well known to pose a higher risk for any surgical intervention, particularly in heart surgery [1–5, 11, 17]. Why is obesity a problem? On one hand, obesity increases the likelihood of non-insulin or insulin-dependent diabetes mellitus, which disrupts wound healing tendency. On the other hand, we are of the opinion that sternal infections in obese patients could be caused by superficial wound healing problems due to the tension of the suprasternal tissue. Diabetic female patients who have large breasts are prone to this complication. Prophylactic sternal reinforcement [11] and/or use of retention sutures [19] can reduce the risk of wound complication. Early diagnosis of any infection is crucial. The earlier the diagnosis, the greater the chances of bone preservation. Meticulous surgical technique (minimization of electrocautery and/or bone wax usage, use of sclerotized IMA grafts, effective bleeding control), retention sutures, and adequate management of blood sugar level in the perioperative period can reduce the incidence of SSWI or DSWI [10, 11, 16–19]. In the event of DSWI and SSWI, we usually use bilateral musculocutaneous advancement flaps [9] to cover the dehiscence following wound pre-treatment by NPWT [7, 8]. This is a reproducible technique that gives an acceptable cosmetic result. Treatment of SSWI is not well established, and there is no gold standard method. The wound may heal secondarily after many wound dressing changes and/or minor NWPT system usage (PICCO). But this can also cause prolonged wound healing with a long-lasting AB treatment and a longer hospital stay or repeated returns to the outpatient care system [10, 11]. For our patient, the first failed plastic surgery intervention was the main problem, and the secondary tissue defect was too large to cover it in a simple manner. Management of these patients is always difficult and tissue ischemia should be considered an additional factor in such complex reconstructions. As a result, a personalized reconstructive plastic surgery intervention using not only bilateral but also rotational and abdominal advancement flaps [19–21] was necessary.

## Conclusion

Indications for major cardiac surgery, especially in the case of obese patients, are always questionable. Over the past 10 years, we have changed our practices for obese and co-morbid patients. Minimally invasive surgery is now considered the approach of the future, and this is also true for cardiac surgery [22, 23]. Revascularization can be performed via mini thoracotomy to avoid full sternotomy. The LAD is the main and most important coronary artery. MIDCAB (Minimally Invasive Direct Coronary Artery Bypass) grafting is a technique for left ventricular anterior wall revascularization using the LIMA for the LAD and/or diagonal artery as a bypass graft. The procedure can be performed through a left anterior mini thoracotomy. This is most often performed when PCI is inadvisable (complex proximal lesions), unsuccessful or not possible (occluded LAD), or as an alternative procedure in obese patients [23]. More than 10 years after this treatment, a hybrid procedure (MIDCAB + PCI) should be considered. In our practice, this was the first and only case where we had to cover a dehiscence of this size and develop a complex procedure. In the case of DSWI or SSWI, the experience and decision-making methodology of the surgeon is crucial for this type of surgery.
